# Tracking and fixed ranking of leukocyte telomere length across the adult life course

**DOI:** 10.1111/acel.12086

**Published:** 2013-05-27

**Authors:** Athanase Benetos, Jeremy D Kark, Ezra Susser, Masayuki Kimura, Ronit Sinnreich, Wei Chen, Troels Steenstrup, Kaare Christensen, Utz Herbig, Jacob von Bornemann Hjelmborg, Sathanur R Srinivasan, Gerald S Berenson, Carlos Labat, Abraham Aviv

**Affiliations:** 1 Geriatric Service, Nancy University HospitalNancy, 54511, France; 2 Inserm U1116, Faculty of Medicine, Université de LorraineNancy, 54500, France; 3 The Hebrew University–Hadassah School of Public Health and Community MedicineEin Kerem, Jerusalem, Israel; 4 Department of Epidemiology, Mailman School of Public Health, Columbia UniversityNew York, NY, 10032, USA; 5 New York State Psychiatric InstituteNew York, NY, 10032, USA; 6 The Center of Human Development and Aging, University of Medicine and Dentistry of New Jersey, New Jersey Medical SchoolNewark, NJ, 07103, USA; 7 Center for Cardiovascular Health, Tulane UniversityNew Orleans, LA, 70118, USA; 8 Epidemiology and Statistics Units, Institute of Public Health, Danish Twin Registry and Danish Aging Research Center, University of Southern DenmarkOdense, Denmark; 9 Department of Microbiology and Molecular Genetics, University of Medicine and Dentistry of New Jersey, New Jersey Medical SchoolNewark, NJ, 07103, USA

**Keywords:** human, leukocytes, longitudinal, Southern blots, telomeres

## Abstract

Short leukocyte telomere length (LTL) is associated with atherosclerosis in adults and diminished survival in the elderly. LTL dynamics are defined by LTL at birth, which is highly variable, and its age-dependent attrition thereafter, which is rapid during the first 20 years of life. We examined whether age-dependent LTL attrition during adulthood can substantially affect individuals’ LTL ranking (e.g., longer or shorter LTL) in relation to their peers. We measured LTL in samples donated 12 years apart on average by 1156 participants in four longitudinal studies. We observed correlations of 0.91–0.96 between baseline and follow-up LTLs. Ranking individuals by deciles revealed that 94.1% (95% confidence interval of 92.6–95.4%) showed no rank change or a 1 decile change over time. We conclude that in adults, LTL is virtually anchored to a given rank with the passage of time. Accordingly, the links of LTL with atherosclerosis and longevity appear to be established early in life. It is unlikely that lifestyle and its modification during adulthood exert a major impact on LTL ranking.

## Introduction

In the general population, short telomere length (TL) in leukocytes is associated with increased risk of atherosclerosis and predicts diminished survival in the elderly (Aviv, 2012). In addition, telomeropathies are typically presented with short leukocyte telomere length (LTL). These include rare monogenic diseases such as dyskeratosis congenita, which stems from catastrophic mutations in telomere-regulating genes, and more common diseases, for example, pulmonary fibrosis and some forms of aplastic anemia (Armanios, 2009; Calado … Young, 2009; Walne *et al*., 2012). The question that follows is to what extent can the interindividual variation in age-dependent LTL attrition during adulthood explain the wide range of LTL among persons of similar age and the associations of LTL with atherosclerosis and longevity in the population at large. Smoking (Valdes *et al*., 2005; Vasan *et al*., 2008; Mirabello *et al*., 2009; Du *et al*., 2012), high body mass index (BMI; Valdes *et al*., 2005), and sedentary life style (Cherkas *et al*., 2008; Du *et al*., 2012*)* have been shown in some studies to be associated with a shorter LTL. Because reverse causality, for example, a short LTL drives people to smoke, is unlikely, these and other unhealthy forms of lifestyle might accelerate the rate of age-dependent LTL attrition. Although a number of longitudinal studies, ranging from 6 months to 13 years (Gardner *et al*., 2005; Aviv *et al*., 2009; Ehrlenbach *et al*., 2009; Nordfjäll *et al*., 2009; Farzaneh-Far *et al*., 2010; Chen *et al*., 2011; Svenson *et al*., 2011; Kark *et al*., 2012), have been performed with the goal of measuring the individual’s rate of LTL attrition, they have not directly addressed the fundamental question of whether LTL attrition during adulthood can substantially affect individuals’ LTL ranking (e.g., longer or shorter LTL) in relation to their peers. In addition, commercial entities have been promoting the measurements of LTL by suggesting that lifestyle changes and behavioral modifications (e.g., regular exercise, stopping smoking, reducing weight) might help individuals with a short LTL manage their ‘biological aging’ by slowing down their rate of LTL attrition (Leslie, 2011). For these reasons, we examined the tracking and ranking of LTL in subjects from four longitudinal studies that jointly covered 6 decades of adult life. Our conclusion is that the overwhelming majority of individuals maintain their LTL ranking during adulthood, that is, their LTL ranking is ostensibly fixed prior to adulthood.

## Results

### Subject characteristics

Leukocyte telomere length was measured on two occasions, at baseline and follow-up examinations in 1156 adult (44% women) in four studies: in Israel, the Jerusalem Lipid Research Clinic (LRC; Kark *et al*., 2012); in the USA, the Bogalusa Heart Study (BHS; Aviv *et al*., 2009); in France, the Evolution de la Rigidite Arterielle (ERA; Benetos *et al*., 2001); and in Denmark, the Longitudinal Study of Aging Danish Twins (LSADT; Bathum *et al*., 2001). The mean ages at the baseline examination and the duration of follow-up for participants from the four groups studied were as follows: LRC, 30 years/13 years; the BHS, 31 years /12.4 years; the ERA, 58 years/9.5 years; and the LSADT, 75 years /10.8 years (Table 1). Variation in the mean LTL at baseline and follow-up between the study samples reflected the age differences of their participants. The average rate of LTL attrition ranged between 23.6 and 31.2 base pairs (bp) per year and was not dependent on the age of the cohort (Table 1). Overall, participants from the LRC, BHS, and ERA showed a higher mean BMI at follow-up versus baseline examinations, while the elderly participants of the LSADT displayed the opposite trend. In all groups, the percentage of smokers was lower at follow-up than at the baseline examinations.

**Table 1:** Characteristics of the 4 cohorts

Cohorts					
Parameter	All	LRC	BHS	ERA	LSADT
*N*	1156	620	271	185	80
Females %	44	33	66	33	70
Age (years)					
Baseline	38 ± 15	30 ± 1	31 ± 5	58 ± 10	75 ± 2
Follow-up	50 ± 14	43 ± 1	43 ± 4	68 ± 10	86 ± 2
Follow-up duration	12.1 ± 1.7	13.0 ± 0.8	12.4 ± 1.8	9.5 ± 0.5	10.8 ± 0.0
LTL (kb)					
Baseline	7.06 ± 0.81	7.34 ± 0.67	7.22 ± 0.73	6.45 ± 0.56	5.85 ± 0.60
Follow-up	6.73 ± 0.77	7.00 ± 0.63	6.83 ± 0.72	6.23 ± 0.54	5.51 ± 0.64
LTL attrition (bp/year)	27.3 ± 16.8	25.7 ± 14.8	32.2 ± 17.8	23.6 ± 15.6	31.2 ± 24
BMI (Kg/m^2^)					
Baseline	25.7 ± 4.8	24.8 ± 3.7	27.0 ± 6.7	26.5 ± 4.2	26.5 ± 4.1
Follow-up	27.6 ± 5.4	27.2 ± 4.6	29.8 ± 7.3	26.8 ± 4.3	24.9 ± 3.7
Difference	1.88 ± 3.14	2.36 ± 2.62	2.76 ± 3.73	0.33 ± 1.66	−1.60 ± 3.62
Smoking (%)					
Baseline	31	37	31	15	23
Follow-up	23	28	22	10	11

### Tracking

**Figure 1:**
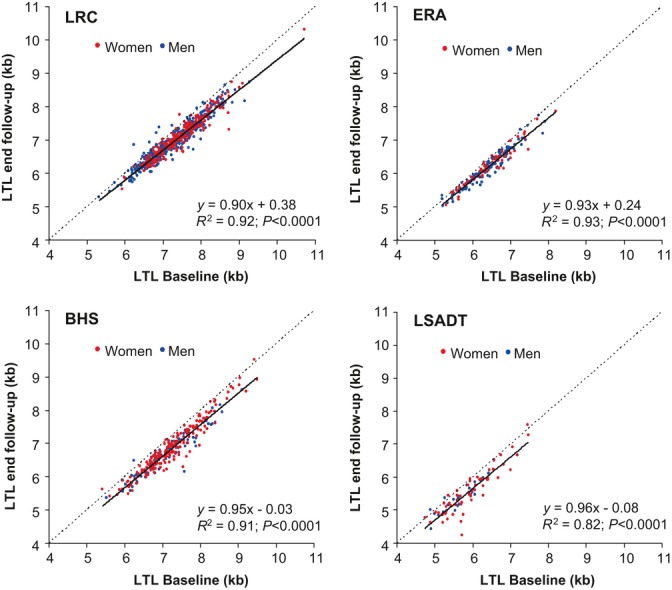
Tracking of leukocyte telomere length (LTL) between baseline and follow-up examinations. Correlation between baseline LTL and follow-up LTL in the Jerusalem Lipid Research Clinic (LRC), the Bogalusa Heart Study (BHS), the Evolution de la Rigidite Arterielle (ERA), and the Longitudinal Study of Elderly Danish Twins (LSADT). The dotted lines are the identity lines, while the continuous lines are the linear regressions of the data. The *R*^2^ (based on Pearson correlations) ranged from 0.82 for the LSADT to 0.93 for the ERA.

Leukocyte telomere length showed robust tracking in the four cohorts, such that individuals with a relatively longer (or a shorter) LTL at baseline also showed a relatively longer (or a shorter) LTL at follow-up (Fig. 1). The Pearson correlations were as follows: LRC, *r* = 0.96; BHS, *r* = 0.95; ERA, *r* = 0.96; and LSADT, *r* = 0.91. The correlation for the LSADT significantly differed from those of the other three cohorts (*P* < 0.05).

### Ranking

**Figure 2:**
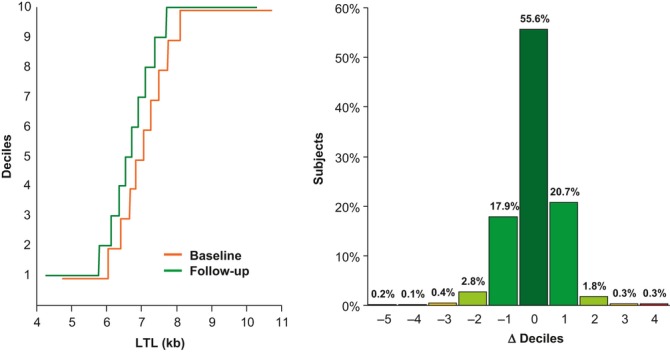
Ranking of leukocyte telomere length (LTL) by deciles for the baseline and follow-up examinations (left panel) and the percentage of subjects experiencing change (Δ) in decile rank at follow-up examination (right panel). Data are from all cohorts jointly. Left panel shows shift to a shorter LTL between baseline and follow-up examinations for each decile. Right panel shows the distribution of Δ between baseline and follow-up examinations. Negative sign denotes a downward shift in ranking, while positive sign indicates an upward shift in ranking. Additional data regarding individual cohorts are provided online in Supplementary Information, Fig. S1, and Table S2 (Supporting information).

**Figure 3:**
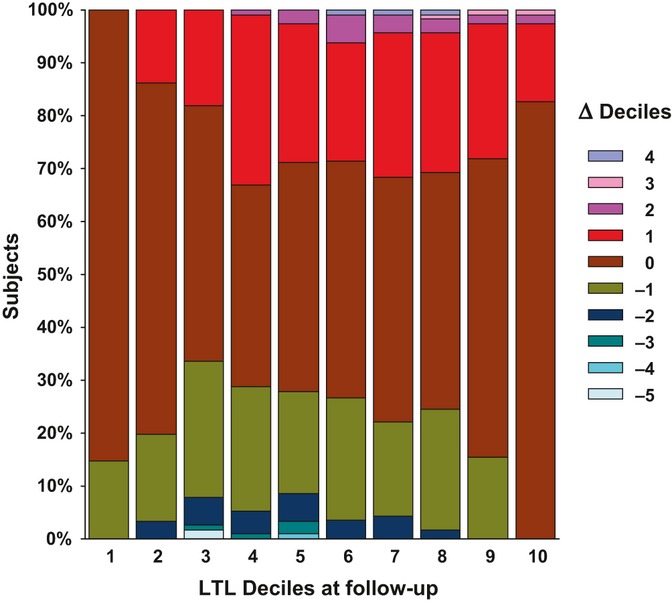
The percentage of subjects in each decile experiencing change (Δ) in rank at follow-up examination. Data are from all cohorts jointly. The following are illustrations of the display: consider decile # 1 (the lowest decile), 14.7% of individuals ranked in this decile at follow-up were ranked in decile #2 at baseline (i.e., one decile downward shift). Now consider decile 2, 16.4% of individuals ranked in this decile at follow-up were ranked at baseline in decile #3 (i.e., one decile downward shift), 3.4% were ranked in decile # 4 (i.e., two deciles downward shift), and 13.8% in decile #1 (i.e., one decile upward shift). The same principle holds across all deciles.

To further examine tracking in the context of the ranking of the individual’s LTL among his/her peers, we divided the LTL distributions at baseline and follow-up examinations into deciles (Fig. 2, left panel) and examined LTL rank changes in the 4 cohorts combined by decile for the entire LTL distribution (Fig. 2, right panel) and for each decile (Fig. 3, Table S1). These data were also examined separately for each of the four cohorts (Fig. S1 and Table S2). In addition, we also examined LTL rank changes by subdividing the LTL distributions at baseline and follow-up examinations into 0.5-kb increments and examined rank changes accordingly in the four cohorts combined (Fig. S2 and Table S3).

Shifts in LTL ranking by one decile up or down are expected to be quite common between baseline and follow-up examinations, that is, because individuals at the interface between two LTL deciles can readily oscillate up/down by one decile, even in the presence of tiny differences in their age-dependent LTL attrition and small measurement error of LTL. Thus, a key appraisal for change in LTL ranking is a ≥ 2 decile shift between the baseline and follow-up examinations.

Jointly, for all deciles and study cohorts, individuals largely maintained their ranks between baseline and follow-up examinations with 55.6% unchanged, 38.6% showing an increase/decrease of one decile (20.7% up/17.9% down), and only 5.9% showing a change of 2 or more deciles in either direction (2.4% up/3.5% down) (Fig. 2, right panel). Thus, 94.1% (95% confidence interval of 92.6–95.4%) showed no change in rank or a change of 1 decile.

When changes in LTL ranking were analyzed by LTL deciles at follow-up, there was a striking stability in the ranking of individuals in the 1st and 2nd deciles, that is, the lower range of the LTL distribution and in the 9th and 10th deciles, that is, the higher range of the LTL distribution (Fig. 3, Table S1). Individuals whose LTL was in the midrange of the distribution also showed stability in their ranking, because only infrequently they experienced a ≥ 2 decile shift between the baseline and follow-up examinations.

Similar results were observed when each cohort was analyzed separately (Fig. S1 and Table S2), and when the baseline and follow-up LTL distributions were subdivided by 0.5-kb increments: 37.4% of the subjects did not change their rank and 57.8% changed rank by only 0.5 kb (Fig. S2 and Table S3).

### The effects of age, sex, smoking, and BMI on ranking

The determinants of the LTL ranking at the follow-up examination were evaluated in a model that included baseline LTL ranking, age, sex, smoking (yes/no), and BMI. LTL ranking at baseline examination was the most important ranking determinant at follow-up examination (regression coefficient (RC) = 0.96 ± 0.01 (SEM), *R*^2^ = 91.3%, *P* < 0.0001). Sex (RC = −0.11 ± 0.05, *R*^2^ = 0.03%, *P* =0.04) and smoking at baseline (RC = −0.15 ± 0.05, *R*^2^ = 0.06%, *P* =0.005) showed minor but statistically significant effects. Age and BMI at baseline exerted no significant effect on ranking at the follow-up examination.

We further examined the effect of change in smoking status on LTL attrition and ranking between baseline and follow-up examinations. As the periods between the two examinations were not identical among participants within a given study and across studies, we also examined the impact of smoking status on LTL shortening with adjustment for follow-up years (Table 2). Overall, smoking was associated with slightly heightened LTL attrition between baseline and follow-up. It also was associated with a small downward shift in ranking between the baseline and follow-up examinations. We observed no significant effect of change in BMI on LTL attrition or ranking in this study.

**Table 2:** The association of change in smoking status during follow-up with change in LTL

ANOVA *P*-value*			0.004	0.006	0.057
Baseline	Follow up	*N*	Δ LTL (kb)	Δ LTL/year (bp)	Δ Decile LTL
No	No	750	0.317 ± 0.007	26.2 ± 0.6	0.053 ± 0.031
No	Yes	45	0.352 ± 0.030	28.6 ± 2.5	−0.015 ± 0.127
Yes	No	145	0.380 ± 0.017**	31.3 ± 1.4**	−0.125 ± 0.071
Yes	Yes	216	0.345 ± 0.014	28.1 ± 1.1	−0.077 ± 0.058

Values are age- and sex-adjusted.

LTL, leukocyte telomere length.

* Overall smoking comparison for each outcome.

** *P* <0.05 vs. No-No smoking. No significant difference between the groups Yes-No and Yes-Yes. Values are mean ± SD.

## Discussion

Our findings indicate that the individual’s LTL is virtually anchored to a given LTL rank as he/she moves across the adult life course. This is remarkably evident for individuals in the extreme deciles of the distribution. Individuals ranked at the 1st and 10th deciles can shift rank only unidirectionally, that is, upward and downward, respectively. Still, at follow-up, no individual ranked in the 1st decile at baseline exhibited a ≥ 2 decile upward shift and similarly only 1.8% of individuals ranked in the 10th decile at baseline showed a ≥ 2 decile downward shift.

These findings challenge the conventional paradigm that links variation in LTL dynamics during adult life to human aging and aging-related diseases (Aviv, 2012). The basis for this paradigm is that inflammation and oxidative stress are distinctive features of atherosclerosis and aging in general. Chronic inflammation entails an increase in leukocyte turnover, which is perpetuated by pro-inflammatory factors and sustained by increased replication of hematopoietic stem cells (HSCs). As telomerase, the reverse transcriptase that adds telomere repeats to the ends of chromosomes, is largely repressed in somatic cells, including HSCs (Broccoli *et al*., 1995; Chiu *et al*., 1996; Yui *et al*., 1998), more frequent HSC replication would augment the rate of LTL shortening. While inflammation impacts LTL attrition through the number of HSC replications, oxidative stress might interfere with telomere biology, because the G triplets of telomeres, which in mammals comprise repeats of TTAGGG nucleotide sextets, are highly sensitive to the hydroxyl radical (Houben *et al*., 2008).

This prevailing view considers LTL as a biomarker of the cumulative burden of inflammation and oxidative stress. It is based on the premise that LTL attrition proceeds in tandem with the accruing burden of inflammation/oxidative stress and predicts the progression of atherosclerosis. Thus, a shorter LTL reflects a higher cumulative burden of inflammation and oxidative stress, increased atherosclerotic risk, and diminished survival. However, our analysis of LTL dynamics does not support such a paradigm. This analysis suggests that most of the interindividual variation in LTL among adults arises early in life, because the ranking of individuals according to their LTL barely changes across 6 decades of adult life.

A constellation of findings support this supposition, including the following: (i) the range of the distribution of LTL at birth amounts to ∼5000 bp (Okuda *et al*., 2002; Akkad *et al*., 2006); (ii) LTL undergoes extremely rapid attrition early in life in humans (Frenck *et al*., 1998; Sidorov *et al*., 2009; Aubert *et al*., 2012) and other mammals (Baerlocher *et al*., 2007; Benetos *et al*., 2011), and by the age of 20 years, human LTL has shortened by approximately 3000 bp; and (iii) on the average, the rate of LTL shortening during adult life amounts to only ∼ 30 bp/year, although with a wide interindividual variation (as shown in Table 1). It is reasonable to propose therefore that the main determinants of LTL in adulthood are LTL at birth and its attrition during the first 20 years of life. This premise is further supported by the finding that the gap in TL between leukocytes, that is, LTL, and TLs in minimally proliferative tissues such as skeletal muscle and subcutaneous fat is established during the first two decades of the human life course (Daniali *et al*., 2013). Accordingly, in many individuals, a short LTL is an early antecedent of health outcomes in adult life. Although environmental factors and lifestyle might alter the rate of LTL attrition during adult life (Valdes *et al*., 2005; Cherkas *et al*., 2008; Vasan *et al*., 2008; Mirabello *et al*., 2009; Du *et al*., 2012), their overall impact on the individual’s LTL (and his/her ranking across the adult lifespan) is expected to be modest compared with the effects of LTL at birth and its attrition during growth and development. This is displayed in the present study with respect to the effects of smoking. To put the smoking effect in perspective, whether or not statistically significant, on average smoking (at baseline, follow-up or both examinations) was associated with acceleration of the rate of LTL attrition by ∼ 3 bp/year and a downward shift of ∼ 0.1 LTL decile ranking between baseline and follow-up examinations (Table 2). Everything else being equal, and assuming causality, it would take 120 years (the average period of ∼ 12 years of follow-up × 10) of smoking to bring about a downward shift of 1 LTL decile.

That being said, the present study was not primarily designed to explore the impact of lifestyle and environmental factors on LTL tracking and ranking. What is more, the absolute TL might not be the only factor through which telomere biology exerts an impact on human aging and disease. Telomeres do not have to be critically short, or even shorter than the average TL in a given cell, to trigger cellular senescence. As double-stranded DNA breaks in telomeres are irreparable, potentially even long telomeres could become dysfunctional in response to genotoxic stresses such as hydroxyl radicals (Fumagalli *et al*., 2012). This could result in an increased accumulation of senescent cells with dysfunctional telomeres in tissue and consequently facilitate aging and disease development in the absence of significant telomere erosion. Indeed, an aging-associated increase in cells displaying dysfunctional telomeres that, on average, were not shorter compared with other telomeres in the same tissue was observed in postmitotic cells of mice and nonhuman primates (Fumagalli *et al*., 2012). Therefore, even a relatively small amount of telomere attrition during adulthood might tip the balance from health to disease without altering the individual’s ranking.

The low measurement error of LTL by Southern blots of the terminal restriction fragments (TRFs; Kimura *et al*., 2010; Aviv *et al*., 2011), the method used to measure LTL in this study, and a sample size of 1156 individuals bolster the validity of our findings. However, ideally, tracking and ranking of LTL with a view to understanding human LTL dynamics (LTL at birth and its age-dependent attrition) should include repeated measurements of LTL in the same individuals followed from birth onward, covering the entire human lifespan. Nonetheless, based on our analysis of four different cohorts, which together cover a broad segment of the adult lifespan, it is evident that close tracking of LTL in adults is the rule.

A main cause of mortality and morbidity in the elderly is atherosclerotic cardiovascular disease. As the proportion of elderly persons is increasing in most modern societies, the quest to understand the mechanisms whereby LTL dynamics explain some of the interindividual variation in susceptibility to atherosclerosis and in survival is pertinent to population health. However, we suggest that to gain further insight into the LTL-aging nexus in humans, future research should also focus on the mechanisms that fashion LTL at birth and its rate of shortening during infancy and childhood. In this context, a recent study showed that in zebra finches, TL at the age of 25 days strongly predicted lifespan (Heidinger *et al*., 2012).

Finally, there is a compelling reason for TL measurements in diagnosing telomeropathies (Armanios, 2009; Calado … Young, 2009; Walne *et al*., 2012). However, the value of LTL measurements in the general population is uncertain. Commercial entities offer these measurements to physicians and the public under the assumption that individuals with a short LTL must have an accelerated age-dependent LTL attrition and that lifestyle modification, such as smoking cessation and regular exercise, might attenuate the rate of LTL attrition. We do not doubt that healthy lifestyle changes can reduce one’s cardiovascular risk and affect longevity, but argue against the view that these outcomes are mediated primarily through LTL change in adulthood.

## Experimental procedures

### Measurements of LTL

Leukocyte telomere length was measured by Southern blots of the TRFs, as previously described (Kimura *et al*., 2010). Briefly, DNA integrity was verified in all samples. To this end, samples (10 ng each) were resolved on a 1% (wt/vol) agarose gel. The TRFs were generated by 16-hour digestion (37°C) using restriction enzymes *Hinf*I and *Rsa*I. A 0.6% (wt/vol) agarose gel, which allows a greater resolution of shorter telomeres, was used in LTL measurements of the elderly participants in the LSADT. A 0.5% (wt/vol) agarose gel was used in LTL measurements of participants in the LRC, the BHS, and the ERA. Each sample was measured in duplicate (3 μg DNA/run) on different occasions (performed on different gels).

**Figure 4:**
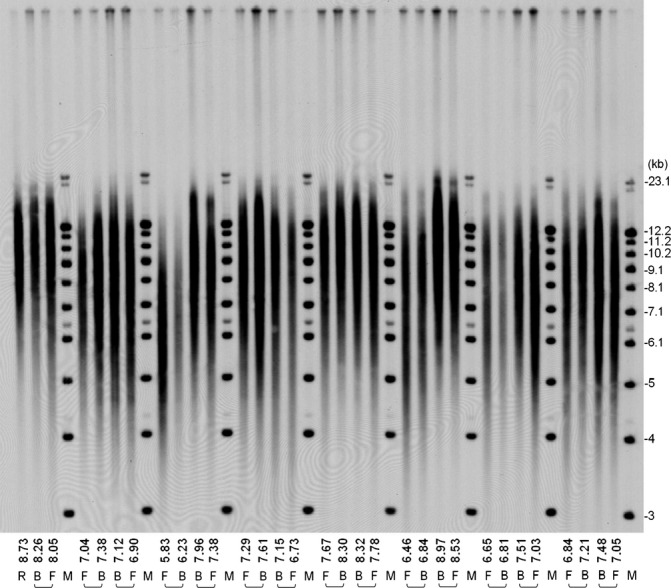
Illustrative gel of Southern blots of the terminal restriction fragments (TRFs) from the Jerusalem Lipid Research Clinic study. A sample of known TRF length serves as an internal reference (R). Eight molecular weight (M) ladders are resolved at intervals across the gel, and the one that is closest to a given sample is used for the computation of the mean TRF for that sample. Baseline (B) and follow-up (F) of the mean TRFs (i.e., leukocyte telomere lengths) are shown (in kb) at the bottom of the lanes.

Personnel in the laboratory that measured LTLs were ‘blind’ with respect to participants. Only numerically coded samples were provided to the laboratory. For longitudinal evaluations, the laboratory was also provided with designations, such A and B, to indicate that samples belong to the same individual. However, such designations varied between baseline and follow-up samples. The baseline and follow-up samples from each individual were run in adjacent lanes, as shown in the illustrative gel (Fig. 4). The interassay coefficient of variations (CV%) was LRC = 2.2%; BHS = 2.4%; ERA = 2.1%; and LSADT = 2.4%.

### Statistical analysis

Pearson correlations and linear regression were used to assess associations between LTL measurements. Deciles of LTL values for individuals in each cohort and for individuals in all cohorts combined were created to measure the individual’s change in rank between the baseline measurement and that at the follow-up. All subjects were also classified by individual changes in 0.5-kb LTL categories. The determinants of the LTL ranking at the follow-up examination were assessed using multiple regression analysis, including in the model the baseline LTL ranking, age, sex, present smoking (yes/no), and BMI. Correlation coefficients (baseline vs. follow-up LTLs) between cohorts were compared with a chi-squared test. One-way ANOVA, followed by a Fisher’s multiple-comparison test, was used to determine the effect of smoking status (adjusted for age and sex) on LTL attrition and change in LTL decile ranking.

## Supporting Information

**Table S1** Percentage of subjects experiencing change (Δ) in decile rank at follow-up examination in the four chorts jointly. **Table S2** Percentage of subjects experiencing change (Δ) in decile rank at follow-up examination in each of the four cohorts. **Table S3** Percentage of subjects experiencing change (Δ) in LTL rank at follow-up examination (each rank corresponding to 0.5 kb) in the four cohorts jointly. **Fig S1** Percentage of subjects experiencing change (Δ) in decile rank at follow-up examination in each of the four cohorts. **Fig S2** Number of subjects experiencing changes in LTL ranking at follow-up (each rank corresponding to a 0.5 kb) in the four cohorts jointly.
