# Supplementary material for: Tracking and fixed ranking of leukocyte telomere length across the adult life course
Source: Aging Cell. 2013 May 27;12(4):615–21. doi: 10.1111/acel.12086 (PMC3798089; doi:10.1111/acel.12086)
Supplement: Supplementary file 1 — Table S1 Percentage of subjects experiencing change (Δ) in decile rank at follow-up examination in the four chorts jointly. Table S2 Percentage of subjects experiencing change (Δ) in decile rank at follow-up examination in each of the four cohorts. Table S3 Percentage of subjects experiencing change (Δ) in LTL rank at follow-up examination (each rank corresponding to 0.5 kb) in the four cohorts jointly. Fig S1 Percentage of subjects experiencing change (Δ) in decile rank at follow-up examination in each of the four cohorts. Fig S2 Number of subjects experiencing changes in LTL ranking at follow-up (each rank corresponding to a 0.5 kb) in the four cohorts jointly. [file acel0012-0615-sd1.doc]

Supplementary Material

**Table S1. Percentage of subjects experiencing change (∆) in decile rank LTL at follow-up examination in the 4 cohorts jointly**

**Δ Deciles**

**Deciles -5 -4 -3 -2 -1 0 1 2 3 4 Total**

1 0.0% 0.0% 0.0% 0.0% 14.7% 85.3% 0.0% 0.0% 0.0% 0.0% **100%**

2 0.0% 0.0% 0.0% 3.4% 16.4% 66.4% 13.8% 0.0% 0.0% 0.0% **100%**

3 1.7% 0.0% 0.9% 5.2% 25.9% 48.3% 18.1% 0.0% 0.0% 0.0% **100%**

4 0.0% 0.0% 0.9% 4.3% 23.5% 38.3% 32.2% 0.9% 0.0% 0.0% **100%**

5 0.0% 0.8% 2.5% 5.1% 19.5% 43.2% 26.3% 2.5% 0.0% 0.0% **100%**

6 0.0% 0.0% 0.0% 3.6% 23.2% 44.6% 22.3% 5.4% 0.0% 0.9% **100%**

7 0.0% 0.0% 0.0% 4.3% 17.9% 46.2% 27.4% 3.4% 0.0% 0.9% **100%**

8 0.0% 0.0% 0.0% 1.8% 22.8% 44.7% 26.3% 2.6% 0.9% 0.9% **100%**

9 0.0% 0.0% 0.0% 0.0% 15.4% 56.4% 25.6% 1.7% 0.9% 0.0% **100%**

10 0.0% 0.0% 0.0% 0.0% 0.0% 82.6% 14.8% 1.7% 0.9% 0.0% **100%**

**Table S2. Percentage of subjects experiencing change (∆) in decile rank at follow-up examination in each of the 4 cohorts.**

**Δ Deciles**

**Cohorts -6 -5 -4 -3 -2 -1 0 1 2 3 4 5 Total**

**LRC** 0.0% 0.0% 0.2% 0.8% 3.5% 20.8% 48.9% 21.5% 3.4% 0.5% 0.3% 0.2% **100%**

**ERA** 0.0% 0.0% 0.0% 1.6% 6.5% 11.9% 54.6% 22.7% 2.7% 0.0% 0.0% 0.0% **100%**

**BHS** 0.4% 0.0% 0.7% 0.4% 5.9% 16.6% 48.3% 22.5% 4.1% 1.1% 0.0% 0.0% **100%**

**LSADT** 0.0% 0.0% 3.8% 1.3% 3.8% 20.0% 38.8% 22.5% 6.3% 3.8% 0.0% 0.0% **100%**

**Table S3. Percentage of subjects experiencing change (Δ) in LTL rank at follow-up examination (each rank corresponds to 0.5 kb) in the 4 cohorts jointly.**

**LTL at Δ LTL (baseline minus follow-up)**

**Follow-up (kb) -1 0 1 2 3 Total**

**4.0 - 4.5** 0.0% 0.0% 0.1% 0.0% 0.1% **0.2%**

**4.5 - 5.0** 0.0% 0.2% 1.1% 0.3% 0.0% **1.6%**

**5.0 - 5.5** 0.1% 1.6% 2.2% 0.3% 0.0% **4.2%**

**5.5 - 6.0** 0.1% 3.5% 6.0% 0.3% 0.0% **9.8%**

**6.0 - 6.5** 0.0% 7.9% 13.3% 0.9% 0.2% **22.2%**

**6.5 - 7.0** 0.2% 10.6% 14.9% 1.4% 0.0% **27.1%**

**7.0 - 7.5** 0.3% 6.6% 11.2% 1.0% 0.1% **19.1%**

**7.5 - 8.0** 0.2% 4.5% 5.9% 0.3% 0.0% **10.9%**

**8.0 - 8.5** 0.0% 1.9% 1.6% 0.1% 0.0% **3.5%**

**8.5 - 9.0** 0.0% 0.6% 0.5% 0.1% 0.0% **1.2%**

**9.0 - 9.5** 0.1% 0.0% 0.0% 0.0% 0.0% **0.1%**

**9.5 - 10.0** 0.0% 0.0% 0.1% 0.0% 0.0% **0.1%**

**Total 0.9% 37.4% 56.9% 4.5% 0.3% 100.0%**

**Figure S1** **Percentage of subjects experiencing change (∆) in decile rank at follow-up examination in each of the 4 cohorts.**

**Figure S2. Number of subjects experiencing changes in LTL ranking at follow-up (each rank corresponding to a 0.5 kb) in the 4 cohorts jointly.**
